# In this issue of *Current Oncology*

**Published:** 2009-12

**Authors:** M. McLean

Welcome, readers, to the last issue of *Current Oncology* for 2009.

Sam Zwenger, in a pre-holiday short communication, develops an interesting (and entertaining) hypothesis about how to limit the risk of oral hpv infection, and presumably a lower risk of subsequent oral cancer, by bogarting (not passing) joints—the joints being in this case weed, rather than beef!

Adjuvant chemotherapy for early breast cancer is known to improve disease-free and overall survival in pre- and postmenopausal women. The importance of maintaining relative dose intensity (rdi) is also well known, but little information is available from routine clinical practice concerning how well dose intensity is maintained with modern and intensive chemotherapy regimens. Dr. Saleem Raza and colleagues report their experience from a single institution (London Regional Cancer Program, London, Ontario), documenting the degree of compliance that they found in a retrospective analysis. Their discovery that the rdi fell below 85% in fewer than 15% of the program’s patients, with older patients being more susceptible, was a gratifying result. In a similar vein, the toxicity of modern colorectal cancer chemotherapy in “real world” non-trial patients has been compared by Dr. Vincent Tam and colleagues (McMaster University and the Juravinski Cancer Centre, Hamilton, Ontario) with the toxicity experienced by the patients that participated in the original studies evaluating those compounds.

Dr. Lucie Lafay-Cousin and her colleagues (Alberta Children’s Hospital, Calgary, Alberta, and Hospital for Sick Children, Toronto, Ontario) lend support to the belief that avoidance of conventional craniospinal irradiation in treatment of very young children with medulloblastoma appears to be associated with a better-preserved neurocognitive profile. Their resulting recommendation is that careful attention to neurocognitive function be an integral part of future studies as treatment in this particular population of infants trends toward lower radiation doses.

This issue of *Current Oncology* also contains three practice guidelines—Cancer diagnostic assessment programs: standards for the organization of care in Ontario, from Dr. Melissa Brouwers and colleagues; Clinician–patient communication: evidence-based recommendations to guide practice in cancer, from Dr. Gary Rodin and colleagues; and a consensus report on the management of early-stage rectal cancer, from the Colorectal Cancer Association of Canada—and, on behalf of the editorial board, I can say that we are pleased that the journal continues to be offered the fruits of these provincial and national efforts for publication.

The journal’s concept of hybrid publication, with some works being published in full in hard copy, and some abstracted (with the full versions online at www.current-oncology.com, where the full issue always appears), continues to evolve. In fact, the editorial board ultimately expects most of the journal’s published manuscripts to appear in hybrid format. And all manuscript abstracts are, of course, submitted to our indexing services—PubMed Central, Excerpta Medica, and so on.

Finally, it is again that time of the year when I have the opportunity to thank all journal contributors, to acknowledge our industry supporters, and to wish everyone involved with the journal a peaceful holiday season and a productive 2010.

